# Proteome of CD9^+^ Plasma Small Extracellular Vesicles Differentiates Stages of HPV-Associated Cervical Neoplasia from Normal Epithelium to Invasive Cancer

**DOI:** 10.3390/life16071181

**Published:** 2026-07-16

**Authors:** Alexander M. Yurin, Natalia L. Starodubtseva, Anna E. Bugrova, Alexey S. Kononikhin, Denis N. Silachev, Vladimir E. Frankevich, Alisa O. Tokareva, Maria I. Indeykina, Ekaterina A. Evtushenko, Alexander A. Yakovlev, Elena A. Mezhevitinova, Eugene N. Nikolaev, Niso M. Nazarova, Vera N. Prilepskaya, Vasiliy S. Chernyshev, Gennadiy T. Sukhikh

**Affiliations:** 1 V.I. Kulakov National Medical Research Center for Obstetrics Gynecology and Perinatology, Ministry of Healthcare of Russian Federation, 117997 Moscow, Russia; yurin988@gmail.com (A.M.Y.); anna.bugrova@gmail.com (A.E.B.); as.kononikhin@gmail.com (A.S.K.); silachevdn@belozersky.msu.ru (D.N.S.); vfrankevich@gmail.com (V.E.F.); a_tokareva@oparina4.ru (A.O.T.); e_mezhevitinova@oparina4.ru (E.A.M.); n_nazarova@oparina4.ru (N.M.N.); v_prilepskaya@oparina4.ru (V.N.P.); v_chernyshev@oparina4.ru (V.S.C.); g_sukhikh@oparina4.ru (G.T.S.); 2 Moscow Center for Advanced Studies, 123592 Moscow, Russia; 3 Emanuel Institute of Biochemical Physics, Russian Academy of Sciences, 119334 Moscow, Russia; mariind@yandex.ru; 4 The Center for Bio- and Medical Technologies, 121205 Moscow, Russia; 5 A.N. Belozersky Research Institute of Physico-Chemical Biology, Lomonosov Moscow State University, 119234 Moscow, Russia; 6 Faculty of Biology, Lomonosov Moscow State University, 119234 Moscow, Russia; evtushenkoea@my.msu.ru; 7 Institute of Higher Nervous Activity and Neurophysiology, Russian Academy of Sciences, 117485 Moscow, Russia; al_yakovlev@ihna.ru; 8 Department of Obstetrics, Gynecology, Perinatology and Reproductology, Institute of Professional Education, Federal State Autonomous Educational Institution of Higher Education I.M. Sechenov First Moscow State Medical University of the Ministry of Health of the Russian Federation, 119991 Moscow, Russia

**Keywords:** cervical cancer biomarkers, small extracellular vesicles, exosomes, proteomics, liquid biopsy

## Abstract

Human papillomavirus (HPV)-associated cervical lesions remain a significant disease burden and minimally invasive blood-based biomarkers could complement cytology and HPV testing. This study aimed to characterize the proteomic composition of plasma-derived CD9^+^ small extracellular vesicles (sEVs) across the morphological spectrum of HPV-associated cervical disease, from histologically normal (NILM) through low-grade (LSIL) and high-grade (HSIL) lesions to invasive squamous cell carcinoma (SCC). Plasma samples from 34 women (NILM, LSIL, HSIL, SCC) were pooled per group, and CD9^+^ sEVs were isolated using an electrochemically controlled immunoaffinity capture method, followed by nanoparticle tracking analysis, transmission electron microscopy, Western blotting, and label-free LC–MS/MS proteomic profiling. The core sEV proteome comprised 258 shared proteins. LSIL showed the most pronounced changes with broad enrichment of complement and coagulation components and acute-phase reactants alongside depletion of immunoglobulin chains and complement C1r-like protein (C1RL). HSIL exhibited few differential proteins, dominated by neutrophil degranulation and retinoid metabolism pathways. SCC demonstrated extensive cargo depletion (22 downregulated proteins) and a nearly sevenfold upregulation of C1RL. Five proteins (including immunoglobulin chains and GPLD1) correlated positively with lesion severity. Pathway analysis consistently implicated platelet activation, lipoprotein remodeling, and insulin-like growth factor signaling. We conclude that plasma CD9^+^ sEVs carry stage-specific proteomic signatures distinguishing HPV-associated cervical lesions, with C1RL emerging as a candidate biphasic marker warranting further validation.

## 1. Introduction

Human papillomavirus (HPV)-associated neoplasms of the anogenital region continue to represent one of the leading causes of cancer incidence and mortality among women worldwide [1,2,3,4]. HPV infection, particularly with high-risk oncogenic types (hrHPV), is the primary etiological factor in the development of squamous intraepithelial lesions and malignancies of the anogenital region. Among these, HPV16 and HPV18 are the most prevalent and potent oncogenic types, responsible for approximately 70% of all cervical cancer cases worldwide. Other high-risk types, including HPV31, 33, 45, 52, and 58, also contribute substantially to the disease burden. The integration of viral DNA into the host genome and the subsequent expression of the E6 and E7 oncoproteins lead to the degradation of tumor suppressors p53 and pRb, triggering a cascade of molecular events that drive malignant transformation [2,3,5,6,7,8,9,10].

Effective control of HPV-associated disease requires diagnostic tools capable of reliably identifying premalignant lesions along the morphological continuum from low-grade squamous intraepithelial lesion (LSIL, CIN1) through high-grade lesion (HSIL, CIN2–3) to invasive squamous cell carcinoma (SCC). Conventional screening relies on cervical cytology and high-risk HPV DNA testing, often used in combination [3]. Cytology offers high specificity (86–100%) but is limited by inter-observer variability and moderate sensitivity, which ranges from approximately 30% to 87% depending on the setting [11]; one large series reported cyto-histological discordance in roughly one in four cases [12]. HPV DNA testing achieves a sensitivity of around 90% for HSIL and invasive disease, but, owing to the transient nature of most HPV infections, has comparatively low specificity, generating a substantial fraction of false-positive results that drive unnecessary downstream procedures [13], and these synchronous anogenital lesions are frequently overlooked because they fall outside the routine remit of both gynaecologists and proctologists. Together, these gaps motivate the search for blood-based, minimally invasive biomarkers that can complement morphology- and virus-based screening.

Liquid biopsy, the analysis of tumour-derived molecular components circulating in peripheral blood and other body fluids, has emerged as a promising avenue [14]. Among its analytes, small tumour-derived extracellular vesicles (sEVs) are receiving increasing attention as carriers of disease-state information. Extracellular vesicles (EVs) are membrane-bound particles released by virtually all cell types and play a central role in intercellular communication by trafficking proteins, lipids, and nucleic acids between donor and recipient cells [15]. According to the current Minimal Information for Studies of Extracellular Vesicles (MISEV) guidelines, sEVs are operationally defined by a hydrodynamic diameter below 200 nm and include vesicles of endosomal origin, commonly termed exosomes, as well as vesicles formed by alternative, non-endosomal mechanisms [16]. The cargo of sEVs is non-randomly packaged and partially reflects the molecular state of the parental cell, rendering them attractive biomarker sources across a range of pathologies [15].

Among sEV cargo classes, surface-associated and luminal proteins are particularly accessible analytes. Mass spectrometry-based proteomic profiling of sEVs isolated directly from patient plasma has been used to identify differentially abundant proteins associated with solid tumors [17,18], and a small but growing body of work has begun to characterize cell-released and plasma sEV proteomes in HPV-associated cervical disease (reviewed in [19,20]; primary studies [21,22]). These studies have implicated processes such as coagulation, complement activation, lipid transport, and cytoskeletal remodelling in cervical neoplastic transformation, and have shown more broadly that tumour-derived EVs can drive matrix remodelling, angiogenesis and immune evasion in solid tumours [23,24,25]. However, considerable heterogeneity exists across studies in the populations sampled, the lesion stages compared, and the methods used to isolate sEVs [19]. The choice of isolation method profoundly influences vesicle yield and the spectrum of co-isolated contaminants, with consequent effects on downstream proteomic readouts [26,27]. Differential ultracentrifugation, the historical reference method, co-purifies abundant non-vesicular plasma proteins [26,28]; size-exclusion chromatography improves purity at the cost of requiring a larger sample volume [29]; and immunoaffinity-based methods provide selectivity for defined vesicle subpopulations at the cost of capturing only marker-positive populations [30]. In this study, the CD9^+^ subpopulation of sEVs was chosen for several key reasons. First, CD9 is one of the most ubiquitously expressed tetraspanins on the surface of sEVs, ensuring a high yield of vesicles from a minimal volume of plasma (only 200 µL) [27]. Second, as demonstrated in our previous work [31], the electrochemical immunoaffinity capture method utilizing anti-CD9 antibodies ensures high specificity and sample purity, effectively minimizing the co-isolation of contaminating plasma proteins compared to conventional ultracentrifugation. Finally, CD9 plays a well-documented role in cell adhesion, motility, and tumor progression, making CD9^+^ sEVs a biologically relevant target for biomarker discovery in oncological diseases.

The aim of the present study was to characterize the proteomic composition of plasma-derived CD9+ small extracellular vesicles across the morphological spectrum of HPV-associated cervical disease, from cytologically and histologically negative controls (NILM) through LSIL and HSIL to invasive SCC, and to identify candidate sEV protein markers that distinguish these stages. Using AS-CD9 immunoaffinity isolation and label-free LC–MS/MS proteomic profiling, we analysed stage-associated alterations at both the individual protein and pathway levels, with the broader goal of supporting the development of minimally invasive blood-based diagnostics for HPV-associated anogenital neoplasia.

## 2. Materials and Methods

### 2.1. Study Design

**Figure 1:**
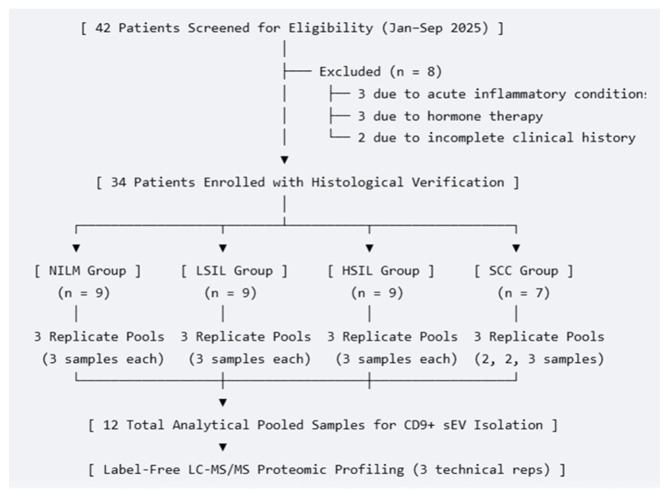
Patient Enrollment and Stratification Flowchart.

This study enrolled 34 women with a mean age of 32.9 ± 7.6 years (median: 32.5 years; range: 23–49 years). All participants received care at the outpatient scientific department of the V.I. Kulakov National Medical Research Center for Obstetrics, Gynecology, and Perinatology (Moscow, Russia) between January and September 2025. This targeted nine-month study window was determined by the operational milestones and strategic enrollment schedule of the pilot phase of our supporting state assignment research grant (Registration No. 12503063216-9). This timeframe allowed for sample saturation of strictly characterized, histologically validated cases processed under identical analytical criteria, fitting the high-throughput discovery nature of the project. Based on biopsy results, patients were stratified into four clinical groups: LSIL, HSIL, SCC, and NILM. The comprehensive patient screening pipeline, clinical cohort breakdown, and subsequent down-stream experimental sample allocation are detailed in Figure 1.

Eligibility criteria included reproductive age (20–49 years), regular menstrual cycles, histologically confirmed diagnoses of NILM, LSIL (CIN I), HSIL (CIN II/III), or squamous cell cervical cancer (SCC) with concurrent presence of high-risk HPV types, and willingness to adhere to the study protocol. Crucially, the control status of patients in the NILM cohort was strictly verified via histological examination of cervical biopsy specimens to rule out any occult or early-stage morphological changes despite active or latent HPV presence. Exclusion criteria encompassed pregnancy, lactation, hormone therapy, acute inflammatory conditions, decompensated renal, hepatic, or pulmonary dysfunction, and psychoneurological disorders.

Sample collection was scheduled during the follicular phase of the menstrual cycle (days 1–5). All 34 participants provided written informed consent. The study received approval from the Ethics Committee of the V.I. Kulakov National Medical Research Center for Obstetrics, Gynecology, and Perinatology (Protocol No. 1, dated 30 January 2025). All procedures adhered to the ethical principles of the Declaration of Helsinki and Good Clinical Practice (GCP) guidelines.

Broad-spectrum HPV genotyping targeting 21 HPV types was conducted using a real-time polymerase chain reaction assay developed by DNK-Technologia (Moscow, Russia). Extended colposcopy was performed using a Leisegang colposcope (Leisegang, Berlin, Germany), following the 2017 International Federation for Cervical Pathology and Colposcopy (IFCPC) terminology (Rio de Janeiro, 2017) [32]. Based on HPV genotyping and colposcopy findings, patients were referred for biopsy.

Cervical biopsy specimens were classified as mild dysplasia (CIN1), moderate dysplasia (CIN2), severe dysplasia (CIN3), or cervical cancer. Histological grading followed the Lower Anogenital Squamous Terminology (LAST) system (2012), where CIN1 corresponds to LSIL, and CIN2/CIN3 correspond to HSIL [33].

### 2.2. Plasma Sample Collection and Processing for sEV Isolation

Peripheral blood was drawn into S-Monovette vacutainer tubes containing EDTA (4.9 mL). Samples were centrifuged at 300× *g* for 20 min at 4 °C to separate plasma from cellular components. The supernatant (plasma) was carefully transferred to fresh tubes, avoiding disruption of the buffy coat (containing leukocytes and platelets), and then subjected to a second centrifugation at 16,000× *g* for 10 min at 4 °C to remove residual cells and platelets. Aliquots of purified plasma (0.5–1 mL) were transferred to 2 mL cryovials, immediately labeled, frozen at −80 °C, and stored until sEV isolation.

For sEV isolation, samples were pooled strictly within each clinical category to create independent biological replicates. No cross-group mixing was performed. Three distinct replicate pools were generated per group as follows: three pools of three samples each for the NILM, LSIL, and HSIL groups; for the SCC group, three pools were formed containing 2, 2, and 3 samples, respectively. Thus, each of the four clinical groups yielded three separate pooled samples, resulting in 12 analytical samples in total for downstream LC-MS/MS proteomic profiling.

### 2.3. Isolation of sEV by Anti-CD9 Immunoaffinity Capture (AS-CD9)

The AS-CD9 method relies on an electrochemically controlled “capture–release” process of vesicles within a microfluidic channel, ensuring high specificity and purity [31]. Selective isolation of CD9+ sEVs was performed from 200 μL of blood plasma using a PMED Separating Cartridge (anti-CD9) and an electrochemical release module (PMED Releasing Unit, AcYtronix, Kloten, Switzerland). Plasma samples were introduced into a microfluidic channel containing microelectrodes functionalized with antibodies against the CD9 surface marker, allowing selective binding of CD9+ sEVs to the electrode surface. After washing the cartridge with phosphate-buffered saline (PBS, pH 7.4) to remove unbound components, sEVs were electrochemically released using the PMED Releasing Unit into deionized Milli-Q water. The resulting sEV fraction was used for subsequent analyses.

### 2.4. Nanoparticle Tracking Analysis (NTA)

sEV concentration and hydrodynamic size distribution were determined using a Nanosight LM10 instrument (Malvern Panalytical, Malvern, UK). Samples were illuminated with a 405 nm laser (40 mW), and scattered light was recorded with a sensitive sCMOS camera (OrcaFlash 2.8 Hamamatsu C 11440, Hamamatsu Photonics, Hamamatsu, Japan). Before measurement, samples were thawed at room temperature (20 °C) for approximately 5 min and diluted in PBS at ratios from 1:100 to 1:500 to achieve 30–60 particles per field of view. Five 60 s videos were recorded per sample at a camera level of 15 and a frame rate of 25 frames/s. Data were analyzed using Nanosight software version 3.2 to determine particle concentration and size distribution. PBS viscosity was assumed equal to that of deionized water at room temperature (~0.91 cP). Size distribution plots were generated with MATLAB R2022b (MathWorks, Natick, MA, USA).

### 2.5. Western Blotting

Protein samples were separated by 15% SDS-PAGE and transferred to nitrocellulose membranes. Membranes were washed with TNT buffer (0.1% Tween 20 in 20 mM Tris-HCl pH 8.0, 150 mM NaCl) and blocked for 30 min at room temperature in 5% skim milk powder in TNT. Subsequently, membranes were incubated overnight at 4 °C with primary antibodies diluted in 5% nonfat dry milk in TNT under gentle agitation. Primary antibodies used were: anti-CD63 (#52090, Cell Signaling Technology, Danvers, MA, USA) at 1:1000, anti-Alix (E-AB-81456, Elabscience, Wuhan, China) at 1:1000, anti-Flotillin-1 (AB133497, Abcam, Cambridge, UK) at 1:10,000. After five washes with TNT (5 min each), membranes were incubated for 1 h at room temperature in 5% skim milk containing horseradish peroxidase-conjugated secondary antibody (goat anti-rabbit IgG (H + L)-HRP conjugate, #1706515, Bio-Rad, Hercules, CA, USA) diluted 1:10,000. Membranes were washed again five times with TNT and developed using SuperSignal™ West Femto Maximum Sensitivity Substrate (Thermo Scientific, Waltham, MA, USA) according to the manufacturer′s instructions. Immunoreactive bands were visualized with a MicroChemi 4.2 imaging system (DNR Bio Imaging Systems, Modi’in-Maccabim-Re’ut, Israel). Protein concentration was determined by the Bradford assay.

### 2.6. Transmission Electron Microscopy (TEM)

Copper grids (1GC300, PELCO, USA) coated with a carbon-stabilized collodion film were used. A 15 μL drop of undiluted sample was applied to the grid, incubated for 1 min, and excess liquid was removed with filter paper. Grids were then negatively stained with 2% uranyl acetate for 10 s and air-dried. Observations were done using a JEM-1400 transmission electron microscope (JEOL, Tokyo, Japan) equipped with a Quemesa digital camera and iTEM software (version 5.2, build 3554) (Olympus Soft Imaging Solutions GmbH, Münster, Germany). Particle sizes were measured with ImageJ software (version 1.52i, National Institutes of Health, Bethesda, MD, USA).

### 2.7. Preparation of sEV Samples for Proteomic Analysis

For proteomic analysis, sEV pellets were dissolved in a reducing buffer containing 6 M urea and 100 mM Tris-HCl (pH 8.0) and shaken for 30 min at 350 rpm. Dithiothreitol (DTT) was added to a final concentration of 10 mM for sulfhydryl reduction, followed by incubation for 30 min at 37 °C. Alkylation was performed by adding iodoacetamide to a final concentration of 20 mM and incubating in the dark for 30 min at 25 °C. Urea concentration was then reduced to 0.5 M by adding 100 mM Tris-HCl, and trypsin was added at a 1:50 enzyme-to-protein ratio. Samples were incubated for 18 h at 37 °C with shaking at 350 rpm. After hydrolysis, samples were acidified with 0.1% (vol/vol) formic acid. The resulting peptides were purified by solid-phase extraction (SPE), lyophilized, and reconstituted in 0.1% formic acid for mass spectrometry.

### 2.8. Liquid Chromatography–Tandem Mass Spectrometry (LC–MS/MS)

Tryptic peptide fractions were analyzed in three technical replicates using a Dionex Ultimate 3000 nanoLC system (Thermo Fisher Scientific, Waltham, MA, USA) coupled to a timsTOF Pro mass spectrometer (Bruker Daltonics, Billerica, MA, USA). Chromatographic separation employed a fixed-spray C18 capillary column (25 cm length, 75 μm inner diameter, 1.6 μm particle size; Ion Optics, Melbourne, VIC, Australia) at a flow rate of 400 nL/min. A 40 min linear gradient from 2% to 37% solvent B (0.1% formic acid in acetonitrile) was applied, followed by a 12 min column wash with 90% solvent B and a 15 min re-equilibration with 2% solvent B. Mobile phases consisted of solvent A (0.1% formic acid in HPLC-grade water) and solvent B (0.1% formic acid in acetonitrile). Mass spectra were acquired using the ddaPASEF method with electrospray ionization (capillary voltage 1500 V, dry gas flow 3.0 L/min, source temperature 180 °C). MS and MS/MS spectra were recorded over an m/z range of 100–1700 and an ion mobility range of 0.6–1.6 1/K0 (V·s/cm^2^). Collision energy increased linearly from 20 eV at 1/K0 = 0.6 V·s/cm^2^ to 59 eV at 1/K0 = 1.6 V·s/cm^2^ with a rise time of 100 ms.

### 2.9. Protein Identification

LC-MS/MS data were processed with PEAKS Studio version 11 (BSI, Mississauga, ON, Canada). Mass tolerance for tryptic peptides was 20 ppm, and for fragments 0.05 Da. Searches were performed against the UniProtKB Homo sapiens database with trypsin specified as the enzyme. Carbamidomethylation of cysteine was set as a fixed modification; methionine oxidation as a variable modification. The false discovery rate (FDR) was set at 0.1% for peptide-spectrum matches (PSMs) and 1% for protein groups. Protein identification required at least two unique peptides. To normalize protein levels in sEV samples, LFQ values were calculated in PEAKS based on Total Ion Current (TIC) and robust averages, which eliminated technical variations between LC-MS/MS runs.

### 2.10. Data Analysis and Statistical Methods

For an omnibus comparison across all four study groups (NILM, LSIL, HSIL, SCC), ANOVA was used for continuous variables and Pearson’s chi-square test for categorical variables. When the initial *p*-value was below 0.05, post hoc analyses were performed using pairwise *t*-tests and pairwise chi-square tests with Bonferroni correction.

Proteins were considered reliably identified if they were detected with at least two unique peptides and appeared in at least two out of three pools in at least one group. Quantitative analysis was performed using the intensity-based absolute quantification (iBAQ) approach.

Proteins showing a statistically significant correlation with the degree of cervical lesion across the sequence NILM → LSIL → HSIL → SCC were identified using Pearson’s correlation test, with a significance threshold of *p* < 0.05. Additionally, marker proteins for each clinical group were identified by comparing protein levels in the target group versus the combined levels in the other three groups using ANOVA (*p* < 0.05). Fold changes were calculated as the ratio of the median protein level in the target group to the median level in the other three groups. Malignancy markers were identified by comparing protein levels in the NILM and LSIL groups versus the HSIL and SCC groups using ANOVA (*p* < 0.05). The fold change was calculated as the ratio of the median level in the HSIL + SCC groups to the median level in the NILM + LSIL groups.

Marker proteins identified for each group were subjected to Reactome pathway enrichment analysis based on a hypergeometric distribution. Pathways with a Benjamini–Hochberg-adjusted *p*-value < 0.05 were considered statistically significantly enriched. Significantly enriched pathways were grouped according to similarity determined by the Jaccard index and hierarchical clustering using Ward’s method.

Statistical analysis and data visualization were performed using R scripts in RStudio (version 2023.09.1) with R version 4.5.3 [34,35]. The following R packages were used: fmsb 0.7.6 [36], VennDiagram 1.8.2 [37], ClusterPro-filer 4.18.4 [38], and ReactomePA 1.54.0 [39]. Data visualization additionally employed ggplot2 4.0.2 [40], ggtree 4.0.5 [41], enrichplot 1.32.0 [42] and ComplexHeatmap 2.26.1 [43].

## 3. Results

### 3.1. Clinical Data

The mean age of patients in the SCC group (40.7 ± 5.0) was statistically significantly higher compared to the HSIL (32.9 ± 7.3; *p* = 0.04), LSIL (28.4 ± 4.7; *p* = 0.003), and NILM (31.3 ± 7.0; *p* = 0.01) groups. The detection rate of HPV type 16 was also higher in the SCC group compared to the LSIL and NILM groups (*p* = 0.03). Furthermore, patients with high-grade precancerous and malignant cervical lesions (HSIL and SCC) had a higher number of sexual partners compared to the NILM and LSIL groups. At the same time, the groups were comparable with respect to weight (*p* = 0.91), height (*p* = 0.51), body mass index (BMI, *p* = 0.91), age at menarche (*p* = 0.24), and the day of the menstrual cycle on which blood plasma was collected (*p* = 0.24) (Table 1).

### 3.2. Characterization of Blood Plasma sEVs

**Figure 2:**
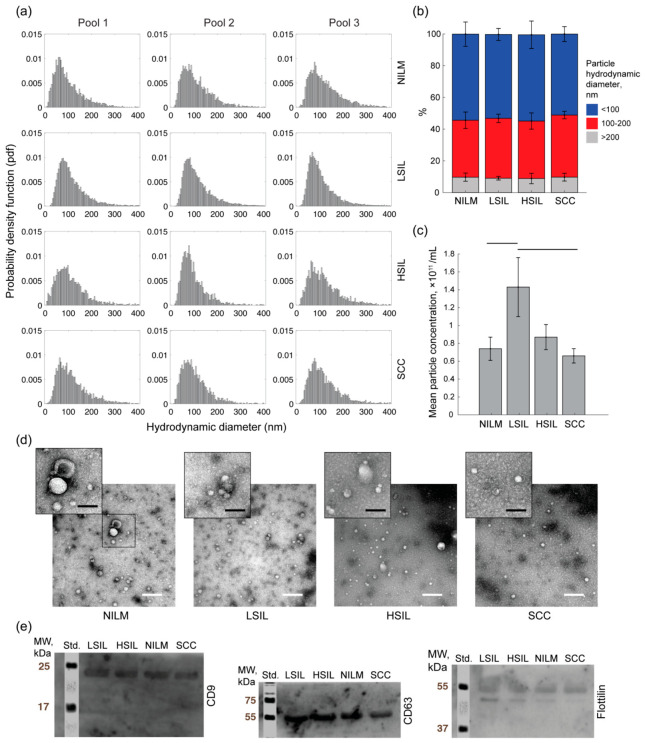
Characterization of sEVs isolated from pooled plasma of LSIL, HSIL, NILM and SCC patients by AS-CD9. (**a**) Hydrodynamic size distribution determined by NTA for each pooled sample, where pdf denotes the probability density function. (**b**) Percentage distribution of particle size range obtained from NTA data. (**c**) Concentration (mean and standard deviation) of isolated vesicles based on NTA. Post hoc analyses were performed using pairwise *t*-tests. Bars signify statistical differences (*p* < 0.05). Statistical test: one-way ANOVA with post hoc pairwise *t*-tests. (**d**) Transmission electron microscopy images of sEVs after isolation (scale bar 200 nm). Insets show magnified portions of the image (scale bar 100 nm). (**e**) Western blot results of known sEV markers.

Small EVs were isolated from 12 samples, each consisting of pooled blood plasma from three LSIL, three HSIL, three SCC, or three NILM patients, using the AS-CD9 method which allows selective isolation of sEVs using immunoaffinity capture with anti-CD9 antibody, a PMED anti-CD9 separation cartridge and electrochemical release by a PMED device. The hydrodynamic size distributions obtained for each pooled sample had minimal variability in mean and median values (Table 2, Figure 2). The mean hydrodynamic diameter was 110 ± 12 nm for NILM, 115 ± 5 for LSIL, 113 ± 12 for HSIL and 113 ± 8 nm for SCC with standard deviation showing variability between three pooled samples. The size distribution was consistent with the results obtained from a previous study [27].

The majority (~90%) of particles characterized by NTA had a hydrodynamic size < 200 which is in the size range of sEVs. The concentration of CD9+ sEVs in blood plasma of LSIL patients was highest (1.43 ± 0.33 × 10^11^ particles/mL) compared to NILM, HSIL and SCC patients with mean particle concentration in the 0.66–0.87 × 10^11^/mL range (Table 2, Figure 2c). TEM analysis showed two particle populations: cup-shaped vesicles and smaller particles without a visible membrane in the size range of exomeres (diameter ~ 35 nm) [38,39] (Figure 2d). Hydrodynamic size exceeds the geometric size of the dried sample [40,41]. Western blot results showed presence of CD9, CD63, and flotillin sEV membrane proteins confirming successful isolation (Figure 2e).

### 3.3. Proteomic Analysis of Plasma sEVs by LC–MS/MS

**Figure 3:**
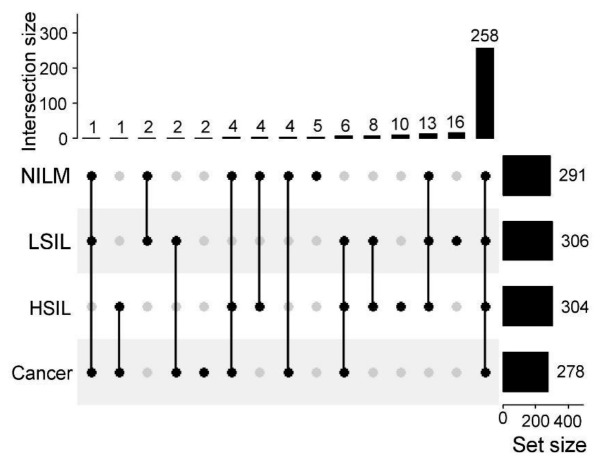
UpSet plot showing the overlap of identified plasma sEV proteins among women with different stages of HPV-associated cervical disease (NILM, LSIL, HSIL, and SCC). Horizontal bars indicate the total number of proteins identified in each group, whereas vertical bars represent the number of proteins shared between the indicated group combinations.

A total of 258 proteins were found to be common to all four study groups, forming the core proteome of plasma sEV (Figure 3, Table S1). Beyond this shared set, each group also displayed a small number of unique proteins. The NILM group had five unique proteins, including structural components and immune-associated proteins such as the cathelicidin antimicrobial peptide (CAMP), the putative macrophage-stimulating 1-like protein (MST1L), and immunoglobulin kappa joining 1 (IGKJ1).

The LSIL group showed 16 unique proteins. Many of these were linked to the ubiquitin–proteasome system, including polyubiquitin chains (UBC, UBB), ubiquitin-ribosomal fusion proteins (RPS27A, UBA52), and the deubiquitinating enzyme OTUD6A. Several actin isoforms (skeletal, cardiac, smooth muscle, and enteric) were also identified, along with proteins involved in the acute inflammatory response, such as C-reactive protein (CRP) and Prostaglandin-H2 D-isomerase (PTGDS). Overall, the LSIL group was enriched in proteins associated with ubiquitination (proteostasis stress), cytoskeletal remodeling, inflammation, and growth signaling—a pattern consistent with early neoplastic transformation.

The HSIL group had ten unique proteins. These were predominantly involved in epithelial structure and adhesion, including Filaggrin (FLG), Desmocollin-1 (DSC1), Desmoglein-1 (DSG1), and several keratins. In addition, the group contained metabolic and stress-related proteins such as Glyceraldehyde-3-phosphate dehydrogenase (GAPDH) and Transferrin receptor protein 1 (TFRC), as well as Cartilage oligomeric matrix protein (COMP) and A-kinase anchor protein 13 (AKAP13), suggesting extracellular matrix remodeling and dysregulation of kinase signaling. Thus, the HSIL group was characterized by proteins reflecting disrupted epithelial architecture, metabolic stress, and matrix signaling—features typical of progression to high-grade dysplasia.

The SCC group had only two unique proteins: Immunoglobulin lambda variable 2-11 (IGLV2-11) and Keratin type II cytoskeletal 72 (KRT72).

### 3.4. sEV Protein Markers of HPV-Associated Cervix Epithelium Transformation

**Figure 4:**
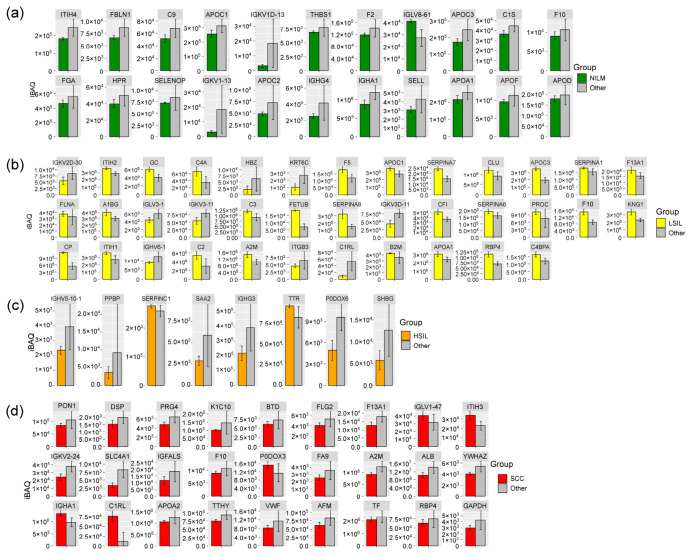
Levels of sEV proteins with significantly different abundances between each target group and the other three combined: (**a**) NILM, (**b**) LSIL, (**c**) HSIL, and (**d**) SCC. Data are presented on the original scale with bars indicating median values and error bars representing the standard deviation. Asterisks denote statistically significant differences as determined by ANOVA with post hoc Bonferroni correction (*p* < 0.05).

Only one protein, Immunoglobulin lambda variable 8-61 (*IGLV8-61*), was significantly more abundant (FC = 1.5, *p* = 0.002) in control (NILM) samples compared to all other groups. In contrast, twenty-one proteins were significantly less abundant (*p* < 0.05) in control samples. These downregulated proteins included multiple apolipoproteins (APOC1, APOC2, APOC3, APOA1, APOF, and APOD), several immunoglobulin chains (IGKV1D-13, IGKV1-13, IGHG4, and IGHA1), coagulation and complement factors (Prothrombin (F2), Coagulation factor X (F10), Fibrinogen α chain (FGA), and Complement component C9 (C9)), protease inhibitors (Inter-α-trypsin inhibitor heavy chain H4 (ITIH4)), and extracellular matrix components (Fibulin-1 (FBLN1), and Thrombospondin-1 (THBS1)), as well as transport proteins (Haptoglobin-related protein (HPR), and Selenoprotein *p* (SELENOP)) and adhesion molecules (L-selectin (SELL)) (Figure 4a). All of these proteins were present at lower levels in sEVs from healthy controls (NILM) and became elevated under pathological conditions (LSIL/HSIL/SCC). This suggests that HPV-associated epithelial transformation leads to increased release of vesicles carrying acute-phase inflammatory proteins, coagulation cascade components, lipid metabolism factors, and immune response mediators into the bloodstream. Their lower abundance in controls simply reflects the absence of active tissue remodeling and inflammation.

During early neoplastic transformation (LSIL), the plasma sEV proteome showed the most pronounced changes (Figure 4b). The 28 enriched proteins included multiple components of the complement and coagulation cascades (Complement C4-A (C4A), Complement C3 (C3), Complement C2 (C2), Complement factor I (CFI), C4b-binding protein α chain (C4BPA), Coagulation factor V (F5), Coagulation factor XIII A chain (F13A1), F10, Vitamin K-dependent protein C (PROC), Kininogen-1 (KNG1)), several protease inhibitors and carrier proteins (Inter-α-trypsin inhibitor heavy chain H2 (ITIH2), Inter-α-trypsin inhibitor heavy chain H1 (ITIH1), A-1-antitrypsin (SERPINA1), Corticosteroid-binding globulin (SERPINA6), A-2-macroglobulin (A2M), Vitamin D-binding protein (GC), Thyroxine-binding globulin (THBG), Retinol-binding protein 4 (RBP4)), and apolipoproteins involved in lipid metabolism (APOC1, APOC3, APOA1), as well as acute-phase and stress-response proteins (Clusterin (CLU), Fetuin-B (FETUB), Ceruloplasmin (CP), A-1B-glycoprotein (A1BG), Β-2-microglobulin (B2M), Filamin-A (FLNA), Angiotensinogen (AGT)). At the same time, nine proteins were significantly less abundant in LSIL samples than in the other groups. These underrepresented proteins included several immunoglobulin variable region chains (IGKV2D-30, IGLV3-1, IGKV3-11, IGKV3D-11, IGHV6-1), a structural component of erythrocytes (hemoglobin subunit zeta (HBZ)), a keratin (KRT6C), Integrin β-3 (ITGB3) involved in cell adhesion and signaling, and a Complement C1r subcomponent-like protein (C1RL).

Thus, LSIL was characterized by sEV enrichment in proteins linked to complement and coagulation activation, protease inhibition, lipid transport, and acute-phase responses. This reflects a systemic inflammatory and hemostatic reaction to early HPV-associated cervical epithelial transformation. Many of the same protein categories were downregulated in controls (NILM) compared to pathological samples, confirming their role in disease progression. At the same time, the marked reduction in immunoglobulin variable chains in sEVs may reflect local suppression of the adaptive immune response during early HPV-associated transformation, allowing the virus and altered cells to evade immune surveillance. The particularly dramatic decrease in C1RL (nearly six-fold) may point to a specific disruption of the classical complement activation pathway.

Interestingly, the sEV proteome in more advanced neoplastic lesions (HSIL) did not show as many pronounced differences as seen in the control and low-grade lesion groups. Only two proteins were significantly more abundant in HSIL samples: Antithrombin-III (SERPINC1), an inhibitor of coagulation, and Transthyretin (TTR), a thyroid hormone and retinol carrier. In contrast, five proteins were significantly less abundant, including an Immunoglobulin heavy variable chain (IGHV5-10-1) and two Immunoglobulin constant chains (IGHG3 and P0DOX6), as well as Platelet basic protein (PPBP) and Serum amyloid A-2 protein (SAA2)—both of which are associated with inflammation and acute-phase responses (Figure 4c). During progression from LSIL to HSIL, there is a weakening of the inflammatory response and humoral immunity—possibly resulting from more effective immune evasion by tumor cells.

Cervical cancer was characterized by a marked decrease in 22 sEV proteins and an increase in 5 others (Figure 4d). The downregulated proteins spanned multiple functional categories, including coagulation factors (F13A1, F10, F9), protease inhibitors and carrier proteins (A2M, Serum Albumin (ALB), Afamin (AFM), Serotransferrin (TF), TTR, Insulin-like growth factor-binding protein complex acid labile subunit (IGFALS), Serum paraoxonase/arylesterase 1 (PON1), Biotinidase (BTD)), structural and cytoskeletal components (Desmoplakin (DSP), Proteoglycan 4 (PRG4), Keratin, type I cytoskeletal 10 (KRT10), Filaggrin-2 (FLG2), 14-3-3 protein zeta/delta (YWHAZ), GAPDH), an apolipoprotein (APOA2), an immunoglobulin chain (IGKV2-24), and the erythrocyte membrane protein (Band 3 anion transport protein (SLC4A1)). In contrast, the five upregulated proteins included two immunoglobulin chains (IGLV1-47 and IGHA1), an immunoglobulin delta heavy chain (P0DOX3), an Inter-α-trypsin inhibitor heavy chain (ITIH3), and a dramatic increase (nearly sevenfold) in C1RL. Thus, SCC was associated with massive downregulation (22 proteins) of diverse protective, transport, structural, and coagulation proteins, which may reflect systemic homeostatic disorganization in late-stage cancer. At the same time, only a small group of five proteins was upregulated, among which C1RL stands out—its nearly sevenfold increase is the most dramatic change in the entire study and may serve as a potential marker of invasive cervical cancer. Notably, this same protein was sixfold decreased in sEV at the low-grade neoplastic stage (LSIL).

**Figure 5:**
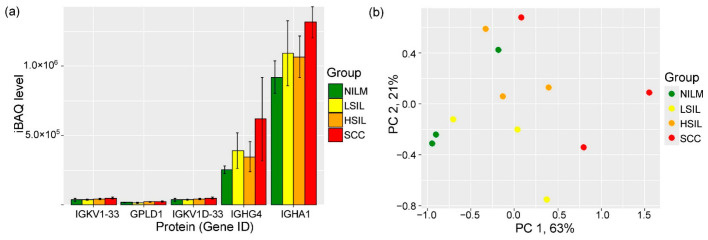
Correlation of sEV protein levels with cervical lesion severity. (**a**) Mean levels of the five sEV proteins that exhibited a statistically significant correlation with disease grade. (**b**) Principal component analysis (PCA) plot of individual samples based on these five proteins. Projection of samples onto the first principal component reflects a progressive increase in lesion severity.

Among the 258 core proteins, five showed a statistically significant (*p* < 0.05) strong positive correlation with the severity of cervical lesions (Table 3, Figure 5a). These included several immunoglobulin constant and variable region proteins (IGKV1-33, IGKV1D-33, IGHA1, and IGHG4), as well as a phospholipase (GPLD1), which is involved in glycosylphosphatidylinositol (GPI) anchor metabolism. Principal component analysis based on these five proteins revealed that samples aligned along the first principal component in order of increasing lesion severity (Figure 5b). Collectively, these five proteins point to a progressive alteration in immune-related signaling: the immunoglobulins represent adaptive humoral responses, while GPLD1 mediates the shedding of GPI-anchored immune receptors from the cell surface.

### 3.5. Pathway Analysis

**Figure 6:**
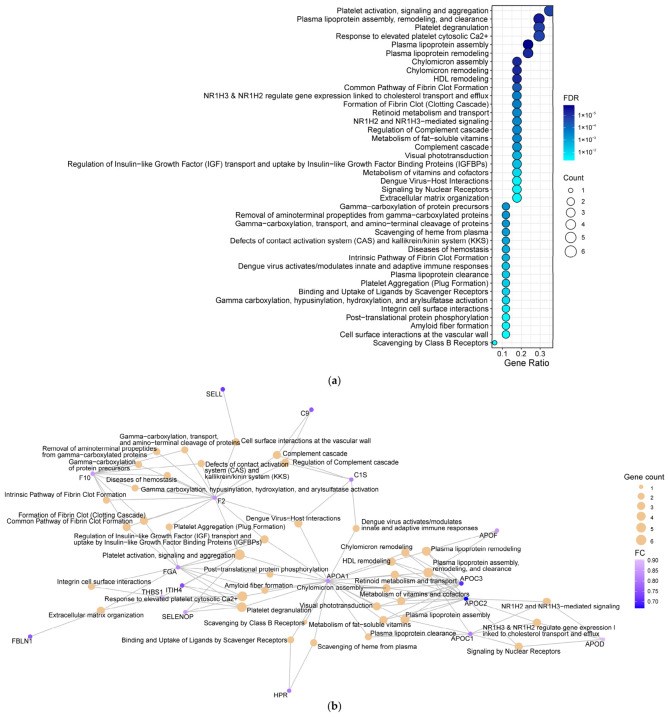

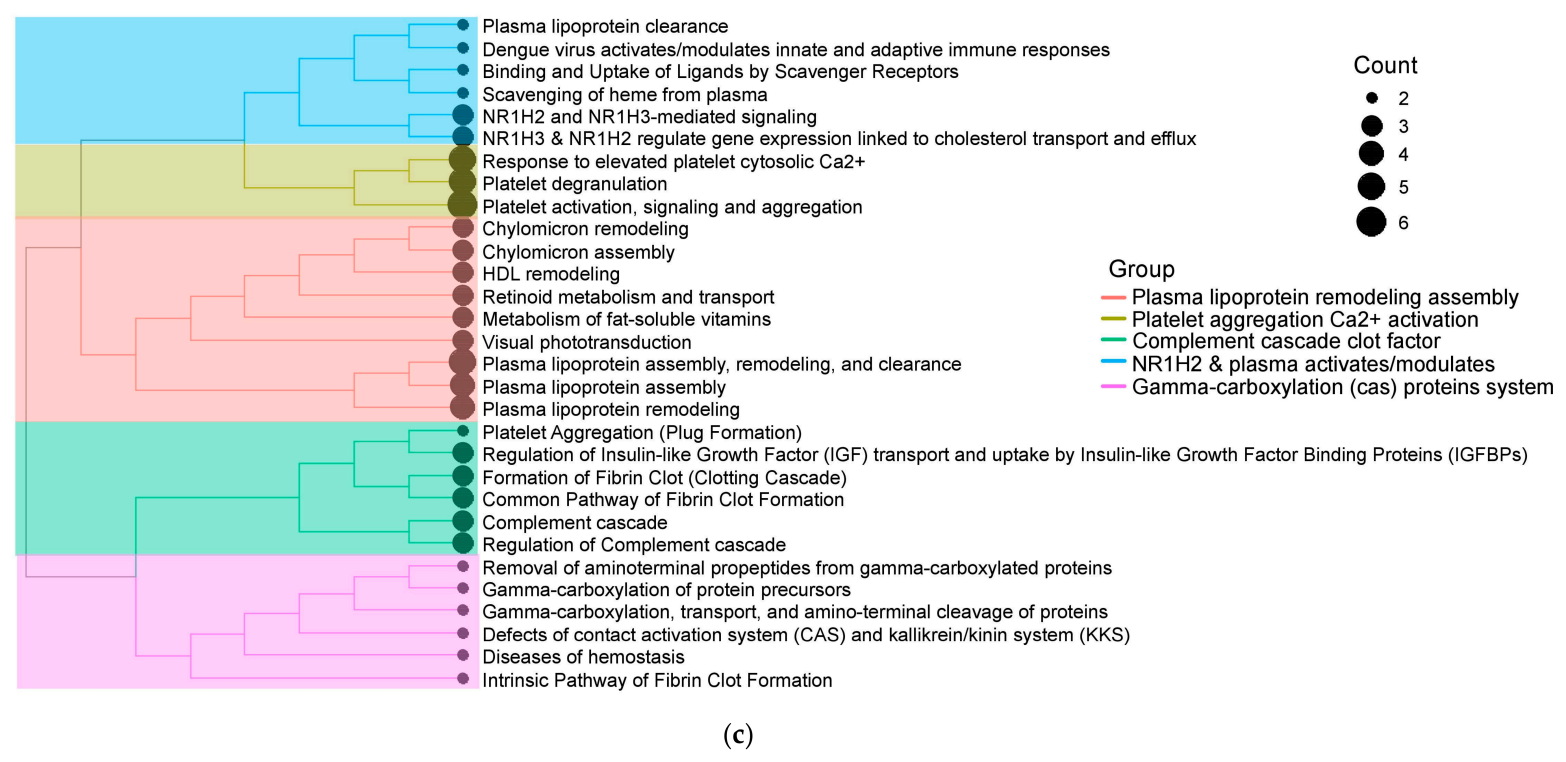
(**a**) Pathways statistically significantly enriched with NILM marker proteins (**b**) Relationship between statistically significantly enriched pathways and the marker proteins they include (**c**) Similarity tree of statistically significantly enriched pathways.

The most pronounced changes in plasma sEV molecular pathways occurred during the transition from normal epithelium (NILM) to HPV-associated cervical lesions (LSIL, HSIL, SCC). A total of 40 pathways were significantly enriched by the 17 down-regulated markers of the NILM group. The pathways containing the largest number of markers were related to platelet activation, signaling, and aggregation; plasma lipoprotein assembly, remodeling, and clearance; and platelet degranulation. Two proteins (APOA1 and prothrombin) were involved in the highest number of pathways (22 and 16, respectively) (Figure 6a,b). Most of these pathways fell into clusters related to plasma lipoprotein remodeling and platelet aggregation (Figure 6c).

The most significant changes in molecular pathways in plasma sEV were observed during the transition from normal (NILM) to HPV-associated cervical epithelial lesions: 40 pathways were identified as statistically significantly enriched with 17 down-regulated NILM markers. Among these, the pathway with the largest number of markers was Platelet activation, signaling and aggregation (proteins ITIH4, THBS1, F2, FGA, SELENOP, APOA1), followed by Plasma lipoprotein assembly, remodeling, and clearance (proteins APOC1, APOC3, APOC2, APOA1, APOF), Platelet degranulation, and Response to elevated platelet cytosolic Ca^2+^ (proteins ITIH4, THBS1, FGA, SELENOP, APOA1). APOA1 and F2 were included in the highest number of pathways (22 and 16, respectively) (Figure 6a,b). Most of the pathways (9) belong to the group of pathways related to plasma lipoprotein remodeling and assembly, while the most significantly enriched pathways belong to the platelet aggregation and Ca^2+^ activation group (Figure 6c).

Twenty-seven pathways were identified as statistically significantly enriched with 20 up-regulated and 1 down-regulated LSIL markers, with the pathways Platelet degranulation, Response to elevated platelet cytosolic Ca^2+^, and Platelet activation, signaling and aggregation (proteins F5, CLU, SERPINA1, F13A1, FLNA, A1BG, KNG1, A2M, ITGB3, APOA1), as well as Post-translational protein phosphorylation and Regulation of Insulin-like Growth Factor (IGF) transport and uptake by Insulin-like Growth Factor Binding Proteins (IGFBPs) (proteins ITIH2, C4A, F5, SERPINA1, C3, PROC, KNG1, CP, APOA1), containing the highest number of markers. APOA1 was included in the highest number of pathways (17) (Figure S1a,b). Most of the pathways (10) belong to the group related to plasma lipoprotein remodeling and assembly, while the most significantly enriched pathways belong to the platelet aggregation and Ca^2+^ activation and insulin groups (Figure S1c).

Nineteen pathways were identified as statistically significantly enriched with 2 up-regulated and 1 down-regulated HSIL markers, with the Neutrophil degranulation pathway (TTR and PPBP) containing the highest number of markers. TTR was included in the highest number of pathways (10) (Figure S2a,b). Based on similarity, the pathways are divided into three groups: Disease metabolism retinoid cycle, degranulation platelet bind Ca^2+^, and Fibrin formation clot factor (Figure S2c).

Twenty-six pathways were identified as statistically significantly enriched with 1 up-regulated and 13 down-regulated cervical cancer (CC) markers, with Platelet activation, signaling and aggregation (proteins F13A1, ITIH3, A2M, ALB, YWHAZ, VWF, TF), Platelet degranulation, and Response to elevated platelet cytosolic Ca^2+^ (proteins F13A1, ITIH3, A2M, ALB, VWF, TF) containing the highest number of markers. APOA2, F10, TTR, RBP4, VWF, and F9 were included in the highest number of pathways (9 for the first two proteins and 8 for all others) (Figure S3a,b). The pathway group related to Disease metabolism retinoid cycle is the largest (8 pathways), while the most significantly enriched pathways belong to the platelet aggregation and Ca^2+^ activation group (Figure S3c).

## 4. Discussion

In this study, the proteome of plasma CD9^+^ sEVs was profiled across the full morphological continuum of HPV-associated cervical neoplasia using a low-volume immunoaffinity capture method validated previously [31]. The isolated vesicles were physically similar across all groups, exhibiting a hydrodynamic diameter of 110–115 nm, a cup-shaped morphology by TEM, and positive staining for CD9, CD63, and flotillin-1. However, their protein cargo differed markedly. The most informative features of the dataset resided in cargo composition rather than in vesicle counts or sizes.

One physical parameter that did differ between groups was particle concentration. LSIL samples had approximately twice the mean concentration of NILM, HSIL, or SCC samples (1.43 ± 0.33 vs. 0.66–0.87 × 10^11^ particles/mL; ANOVA *p* = 0.005). This concentration pattern was non-monotonic, with the highest concentration observed at the lowest grade of disease. A plausible interpretation is that productive HPV infection in differentiating keratinocytes drives a burst of vesicle release for inflammatory activation [44], and that this burst attenuates as lesions progress and acquire effective immune evasion [45]. An alternative possibility is that vesicle trafficking in HSIL and SCC becomes increasingly retained within the tumor microenvironment rather than entering systemic circulation; however, this cannot be formally excluded. Furthermore, the age difference between groups may confound interpretation, as SCC patients in this cohort were older and circulating EV abundance is known to vary with age [46]. The use of pooled samples, although appropriate for an exploratory proteomic screen aimed at reducing inter-individual noise and material requirements, makes these considerations more difficult to test rigorously. Any clinical application of the LSIL concentration peak would therefore require validation on individual samples, rather than pooled material, as a first step.

The core proteome identified in this study (258 proteins shared across all four groups) maps reasonably well onto the dominant constituents of plasma sEVs reported in recent reference datasets [21,45]. A clear explanation for this opposing directional change in early lesions is not available; the pattern is consistent with the selective down-packaging of immune-effector cargo (immunoglobulins and complement initiators) into circulating vesicles, a feature described in early HPV-driven immune escape phenotypes [43].

HSIL was, perhaps surprisingly, the quietest stage in the differential analysis, with only two enriched proteins (SERPINC1, TTR) and five depleted proteins (including PPBP, SAA2, and three immunoglobulin chains). Unique-protein analysis revealed several epithelial constituents, namely FLG, DSC1, DSG1, and several keratins, suggestive of a disrupted epithelial barrier. The appearance of these markers in vesicular cargo at the HSIL stage is consistent with paracrine trafficking between cervical tumor cells and stromal fibroblasts, as previously documented for cervical exosomal Wnt2B [21]. Nevertheless, the overall systemic picture is one of a stage at which the vesicular signal becomes more difficult to detect. This likely reflects the well-characterized HPV E6/E7 immune evasion machinery, one component of which (exosomal PD-L1) has been shown to directly suppress T-cell killing of tumor cells [45]. The possibility that vesicle trafficking at this stage becomes increasingly localized cannot be excluded. The pathway analysis, dominated by neutrophil degranulation and retinoid metabolism with TTR as the most pathway-connected protein, is broadly consistent with this interpretation.

SCC was characterized by extensive cargo depletion: 22 downregulated proteins spanning transport, structural, coagulation, and protease-inhibitor categories, alongside a small set of five upregulated proteins. The breadth of downregulation across functionally unrelated categories is consistent with the systemic homeostatic disorganization typically observed in advanced malignancy [47], although age represents a significant confounder (SCC mean 40.7 ± 5.0 years vs. 28.4–32.9 in the comparator groups; *p* ≤ 0.04). Disease-driven and age-driven changes cannot be completely separated. The most striking change in the dataset was the behavior of C1RL: approximately sixfold downregulated in LSIL, then nearly sevenfold upregulated in SCC—a pattern that would be missed in a monotonic analysis. Functionally, C1RL is a serine protease whose best-characterized substrate is prohaptoglobin, which it cleaves in the endoplasmic reticulum [48]. Haptoglobin itself is an acute-phase reactant and a hemoglobin scavenger; therefore, altered C1RL availability is plausibly linked to systemic inflammatory homeostasis. C1RL is also expressed in monocytes and dendritic cells, so changes in its packaging into circulating sEVs could reflect myeloid activation state in addition to, or instead of, hepatic synthesis. The reason for such marked depletion of this enzyme in LSIL followed by a strong rebound in SCC is not immediately apparent from the present data, but this biphasic pattern identifies C1RL as a candidate worthy of follow-up with targeted assays on individual samples.

Five proteins increased monotonically with lesion severity: IGKV1-33, IGKV1D-33, IGHA1, IGHG4, and GPLD1. Principal component analysis based on this panel separated samples along PC1 in order of clinical stage. Notably, three of the immunoglobulin chains had essentially identical correlation coefficients (R = 0.60), suggesting they may not represent independent signals; the effective dimensionality of the panel is therefore likely lower than five. GPLD1 is the more interesting entry. This enzyme cleaves the GPI anchor that tethers a structurally diverse set of cell-surface proteins, and its expression has been reported to increase with malignancy in epithelial cell lines, including HaCaT keratinocytes and A431 epidermoid carcinoma cells, with parallel increases in shedding of GPI-anchored markers such as the urokinase receptor and CA125 [49]. An increase in vesicular GPLD1 from NILM through to SCC could, in principle, contribute to the loss of GPI-anchored receptors from the cervical epithelial surface. However, most circulating GPLD1 is liver-derived, and the present data do not allow apportionment of vesicular GPLD1 between hepatic and lesional sources.

At the pathway level, the same three themes recurred across stages: platelet activation/degranulation/aggregation, plasma lipoprotein assembly and remodeling, and post-translational regulation of insulin-like growth factor signaling. A caveat is warranted regarding the platelet signal. Cancer-associated thromboinflammation is a well-established systemic phenomenon [50], and tumor-derived EVs do interact with platelets [44]; however, residual platelet-derived sEVs are a known contaminant in plasma EV preparations that is not fully removed by a 16,000× *g* spin [51]. A clean separation between genuine tumor-platelet biology and co-isolation artifacts is therefore not possible. The lipoprotein signal (APOA1, APOC1, APOC3, APOA2) carries a related but mechanistically distinct caveat: cancer-associated dyslipidemia is well documented [52] but in CD9-immunoaffinity-captured sEV preparations, apolipoproteins may also be detected as part of the vesicle-associated protein corona or as surface-bound plasma proteins rather than as intrinsic vesicular cargo [51]. Thus, the pathway results may reflect a host-wide response to HPV-associated cervical disease, together with plasma-derived proteins adsorbed onto circulating sEVs, rather than lesion-derived vesicular cargo alone. Some of these proteins may originate from systemic inflammatory and immune responses, including myeloid-cell activation and acute-phase signaling triggered by persistent HPV infection, thereby reflecting organism-level immunological adaptation in addition to local cervical pathology [16].

In the broader context of the cervical sEV literature, existing reviews [19,20] have cataloged candidate cargo molecules but noted the absence of stage-stratified plasma data spanning the full continuum from NILM through LSIL and HSIL to SCC. A recent multiomics study by Yan and colleagues [22] partially addresses this gap and overlaps with the present findings at the pathway level (complement, coagulation, lipid transport), although methodological differences in vesicle isolation and cohort design complicate protein-by-protein comparisons. Beyond cervical disease, plasma EV proteomic stratification has been used to separate triple-negative breast cancer from healthy and benign controls [17] and to identify EV phosphoprotein markers in breast cancer plasma [18]; the broad themes (immunoglobulin, complement, lipid-transport reorganization) recur across these studies. Convergence at the pathway level is encouraging, whereas divergence at the level of individual marker proteins highlights the lack of methodological standardization in plasma EV biomarker research. From a clinical perspective, the stage-specific proteomic signatures identified in plasma CD9^+^ sEVs hold potential as a non-invasive diagnostic triage tool. Currently, high-risk HPV DNA testing suffers from low specificity due to transient infections, leading to substantial patient anxiety and unnecessary colposcopical over-referrals. A blood-based liquid biopsy panel tracking early acute-phase and tissue-remodeling shifts (as seen in LSIL) or the dramatic biphasic rebound of C1RL (indicative of SCC) could complement cytology. This framework could refine patient stratification, helping clinicians monitor low-grade regression versus high-grade progression without relying solely on serial invasive biopsies.

Several limitations deserve explicit mention. First, the sample size was modest (n = 7–9 per group, processed as three pools of 2–3 samples each), which limits statistical power and precludes formal classifier development. Second, a single immunoaffinity method targeting CD9 was used; this approach yields a high-purity, low-volume preparation but captures only the CD9^+^ sEV subpopulation [16]. Third, the SCC group was significantly older than the comparator groups, which reflects the natural age distribution of invasive disease but introduces age confounding into the SCC-specific signature, including the concentration data. Finally, every candidate marker reported here—including the early LSIL concentration peak, the novel biphasic expression pattern of C1RL, and the five severity-correlated proteins—demands targeted verification. Because individual sample volume constraints necessitated a pooled exploratory screen to achieve an adequate vesicle yield, these findings represent an initial discovery phase. Validation in a larger, completely independent cohort utilizing individual patient samples is a mandatory next step, ideally employing high-throughput orthogonal targeted quantification methods such as parallel reaction monitoring (PRM) mass spectrometry or specialized immunoassays.

The proteomic signatures reported here are intended to contribute to a multiomic diagnostic framework integrating vesicular proteomic, lipidomic, and microRNA data from a single liquid biopsy [44]. Pairing the C1RL pattern with vesicular microRNA or lipid-class biomarkers may improve stage discrimination beyond what any single modality can offer.

Taken together, these findings indicate that plasma CD9^+^ sEVs carry stage-specific proteomic signatures capable of distinguishing HPV-associated cervical lesions across the diagnostic continuum. Early dysplasia stands out for broad acute-phase, coagulation, and lipid-transport enrichment together with selective immunoglobulin and C1RL depletion. HSIL is comparatively quiet, consistent with HPV immune evasion at this stage. SCC shows extensive cargo depletion together with a sevenfold rebound of C1RL.

## 5. Conclusions

This study demonstrates that plasma CD9+ sEVs carry stage-specific proteomic signatures capable of distinguishing HPV-associated cervical lesions from normal epithelium to invasive cancer. Importantly, the data reveal that the systemic vesicular response to HPV-associated neoplasia is highly non-linear across disease progression. The most pronounced proteomic reorganization occurred at the LSIL stage and was characterized by broad enrichment of acute-phase reactants, coagulation factors, complement components, and lipid transport proteins, coupled with selective depletion of immunoglobulin chains and the complement-associated protease C1RL. In contrast, HSIL exhibited comparatively muted vesicular alterations, consistent with progressive immune evasion and reduced systemic inflammatory signaling, whereas SCC was marked by extensive cargo depletion together with a striking rebound of C1RL.

Five proteins, including GPLD1 and several immunoglobulin chains, correlated with lesion severity and may represent candidate progression-associated biomarkers. At the pathway level, platelet activation, lipoprotein remodeling, and insulin-like growth factor signaling emerged as recurrent features across disease stages, suggesting that circulating sEV proteomes reflect not only lesion-derived vesicular cargo, but also broader host systemic and immune responses to persistent HPV infection.

Although limited by modest cohort size, pooled-sample design, and age confounding, this exploratory study demonstrates the utility of immunoaffinity-isolated plasma sEV proteomics for detecting biologically meaningful stage-associated patterns that may be difficult to resolve in highly heterogeneous individual plasma samples. The biphasic behavior of C1RL and the severity-correlated protein panel warrant further validation using orthogonal targeted assays and larger individual-sample cohorts. Ultimately, these findings support the integration of vesicular proteomic markers into future multiomic liquid biopsy strategies for minimally invasive diagnosis and monitoring of HPV-associated anogenital neoplasia.

## Figures and Tables

**Table 1:** Demographic and clinical characteristics of patients across the spectrum of HPV-associated cervical disease.

Parameter	NILM (*n* = 9)	LSIL (*n* = 9)	HSIL (*n* = 9)	SCC (*n* = 7)	*p*-Value
Age, years	31.3 ± 7.0	28.4 ± 4.7	32.9 ± 7.3	40.7 ± 5.0	0.002 *p*_N/C_ = 0.01 *p*_L/C_ = 0.003 *p*_H/C_ = 0.04
Weight, kg	64.0 ± 12.8	66.1 ± 8.1	67.3 ± 13.9	62.7 ± 14.2	0.91
Height, cm	166.2 ± 3.8	168.2 ± 3.9	165.8 ± 4.2	165.0 ± 5.6	0.51
BMI, kg/m^2^	23.0 ± 4.4	23.6 ± 2.1	24.6 ± 5.3	22.9 ± 4.3	0.91
Age at menarche, years	13.0 ± 1.7	13.3 ± 1.2	12.1 ± 1.2	12.9 ± 0.7	0.24
Menstrual cycle day at sampling	9.3 ± 1.4	9.4 ± 1.0	9.0 ± 1.2	9.0 ± 1.3	0.82
Family history of cancer, *n* (%)	3 (33%)	3 (33%)	4 (44%)	3 (43%)	1.00
Age at first sexual intercourse, years	20.3 ± 4.7	18.9 ± 2.4	17.3 ± 1.5	18.3 ± 2.1	0.22
Number of sexual partners	2.1 ± 1.3	2 ± 1	4.4 ± 1.4	4.3 ± 1.6	0.002 *p*_N/H_ = 0.002 *p*_N/C_ = 0.004 *p*_L/H_ = 0.002 *p*_L/C_ = 0.004
History of STIs, *n* (%)	0 (0%)	1 (11%)	1 (11%)	2 (29%)	0.41
Smoking, *n* (%)	0 (0%)	0 (0%)	2 (22%)	1 (14%)	0.27
HPV type					
11 type, *n* (%)	1 (11%)	0 (0%)	0 (0%)	0 (0%)	1.00
16 type, *n* (%)	3 (33%)	3 (33%)	5 (56%)	7 (100%)	0.03 *p*_N/C_ = 0.03 *p*_L/C_ = 0.03
18 type, *n* (%)	0 (0%)	2 (22%)	1 (11%)	0 (0%)	0.59
31 type, *n* (%)	0 (0%)	0 (0%)	2 (22%)	0 (0%)	0.23
33 type, *n* (%)	0 (0%)	3 (33%)	1 (11%)	0 (0%)	0.21
35 type, *n* (%)	0 (0%)	1 (11%)	1 (11%)	0 (0%)	1.00
39 type, *n* (%)	2 (22%)	0 (0%)	0 (0%)	0 (0%)	0.23
44 type, *n* (%)	0 (0%)	2 (22%)	0 (0%)	0 (0%)	0.23
51 type, *n* (%)	1 (11%)	0 (0%)	0 (0%)	0 (0%)	1.00
52 type, *n* (%)	0 (0%)	2 (22%)	1 (11%)	0 (0%)	0.59
53 type, *n* (%)	0 (0%)	2 (22%)	1 (11%)	0 (0%)	0.59
56 type, *n* (%)	2 (22%)	2 (22%)	2 (22%)	0 (0%)	0.58
58 type, *n* (%)	0 (0%)	1 (11%)	0 (0%)	0 (0%)	1.00
66 type, *n* (%)	1 (11%)	0 (0%)	0 (0%)	0 (0%)	1.00
68 type, *n* (%)	1 (11%)	0 (0%)	0 (0%)	0 (0%)	1.00

Note: Data are presented as mean ± standard deviation or *n* (%). BMI, body mass index; STI, sexually transmitted infection; NILM, negative for intraepithelial lesion or malignancy; LSIL, low-grade squamous intraepithelial lesion; HSIL, high-grade squamous intraepithelial lesion; SCC, squamous cell carcinoma. *p* values were calculated using one-way ANOVA or Fisher’s exact test, as appropriate. Significant pairwise comparisons are indicated as follows: N, NILM; L, LSIL; H, HSIL; C, SCC.

**Table 2:** Mean and median hydrodynamic diameters of plasma sEVs and particle concentrations with standard deviations from three pooled samples and *p*-values (one-way ANOVA). In cases where the *p*-value was below 0.05, post hoc analyses were performed using pairwise *t*-tests.

	NILM	LSIL	HSIL	SCC	*p*-Value
Mean, nm	110 ± 12	115 ± 5	113 ± 12	113 ± 8	0.95
Median, nm	93 ± 12	96 ± 5	95 ± 11	99 ± 7	0.91
Concentration, ×10^11^/mL	0.74 ± 0.13	1.43 ± 0.33	0.87 ± 0.14	0.66 ± 0.08	0.005 *p*_N/L_ = 0.03 *p*_L/C_ = 0.02

**Table 3:** sEV proteins with a statistically significant correlation with malignancy grade, including corresponding gene names, correlation coefficients (R), confidence intervals (CI), and *p*-values.

Protein	Gene	R	CI	*p*
Immunoglobulin κ variable 1-33	IGKV1-33	0.60	0.037–0.87	0.04
Phosphatidylinositol-glycan-specific phospholipase D	GPLD1	0.63	0.094–0.89	0.03
Immunoglobulin κ variable 1D-33	IGKV1D-33	0.60	0.037–0.87	0.04
Immunoglobulin heavy constant γ 4	IGHG4	0.60	0.046–0.87	0.04
Immunoglobulin heavy constant α 1	IGHA1	0.67	0.16–0.9	0.02

## Data Availability

The data presented in this study are available on request from the corresponding author.

## Supplementary Materials

The following supporting information can be downloaded at: https://www.mdpi.com/article/10.3390/life16071181/s1.

## Abbreviations

The following abbreviations are used in this manuscript:

ANOVA	Analysis of variance
HPV	Human papillomavirus
LSIL	Low-grade squamous intraepithelial lesion
HSIL	High-grade intraepithelial lesion
SCC	Invasive squamous cell carcinoma
CIN	Cervical intraepithelial neoplasia grade
sEVs	Small extracellular vesicles
GPI	Glycosylphosphatidylinositol
C1RL	complement C1r-like protein
IGF	Insulin-like Growth Factor
IGFBPs	Insulin-like Growth Factor Binding Proteins
MISEV	Minimal Information for Studies of Extracellular Vesicles
LC-MS/MS	Liquid chromatography-tandem mass spectroscopy
MW	Molecular weight
TEM	Transmission electron microscopy
CV	Coefficient of variation
DI water	Milli-Q water
FC	Fold change
FDR	False discovery rate
pdf	Probability density function
iBAQ	Intensity-based absolute quantification
NTA	Nanoparticle tracking analysis
PBS	Phosphate-buffered saline
PCA	principal component analysis
PC	principal component
AS-CD9	immunoaffinity capture using anti-CD9 antibodies
rpm	revolutions per minute
