# Supplementary material for: Proteome of CD9+ Plasma Small Extracellular Vesicles Differentiates Stages of HPV-Associated Cervical Neoplasia from Normal Epithelium to Invasive Cancer
Source: Life (Basel). 2026 Jul 16;16(7):1181. doi: 10.3390/life16071181 (PMC13412234; doi:10.3390/life16071181)
Supplement: Supplementary file 1 [file life-16-01181-s001.zip › Supplementary_figures_life-4389219.pdf]

(a)

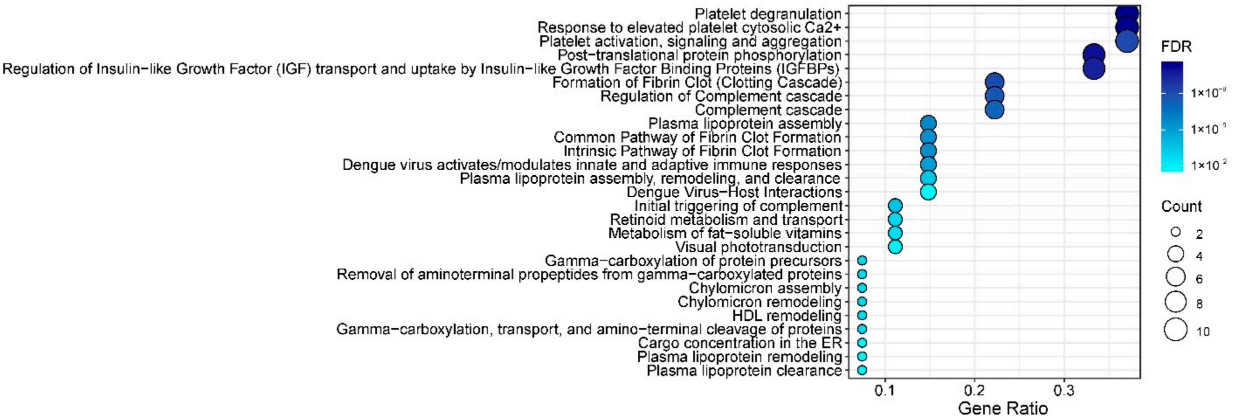
Platelet degranulation
Response to elevated platelet cytosolic Ca2+
Platelet activation, signaling and aggregation
Post−translational protein phosphorylation
Regulation of Insulin−like Growth Factor (IGF) transport and uptake by Insulin−like Growth Factor Binding Proteins (IGFBPs)
Formation of Fibrin Clot (Clotting Cascade)
Regulation of Complement cascade
Complement cascade
Plasma lipoprotein assembly
Common Pathway of Fibrin Clot Formation
Intrinsic Pathway of Fibrin Clot Formation
Dengue virus activates/modulates innate and adaptive immune responses
Plasma lipoprotein assembly, remodeling, and clearance
Dengue Virus−Host Interactions
Initial triggering of complement
Retinoid metabolism and transport
Metabolism of fat-soluble vitamins
Visual phototransduction
Gamma−carboxylation of protein precursors
Removal of aminoterminal propeptides from gamma−carboxylated proteins
Chylomicron assembly
Chylomicron remodeling
HDL remodeling
Gamma−carboxylation, transport, and amino−terminal cleavage of proteins
Cargo concentration in the ER
Plasma lipoprotein remodeling
Plasma lipoprotein clearance
0.1
0.2
0.3
Gene Ratio
FDR
Count
2
4
6
8
10

(b)

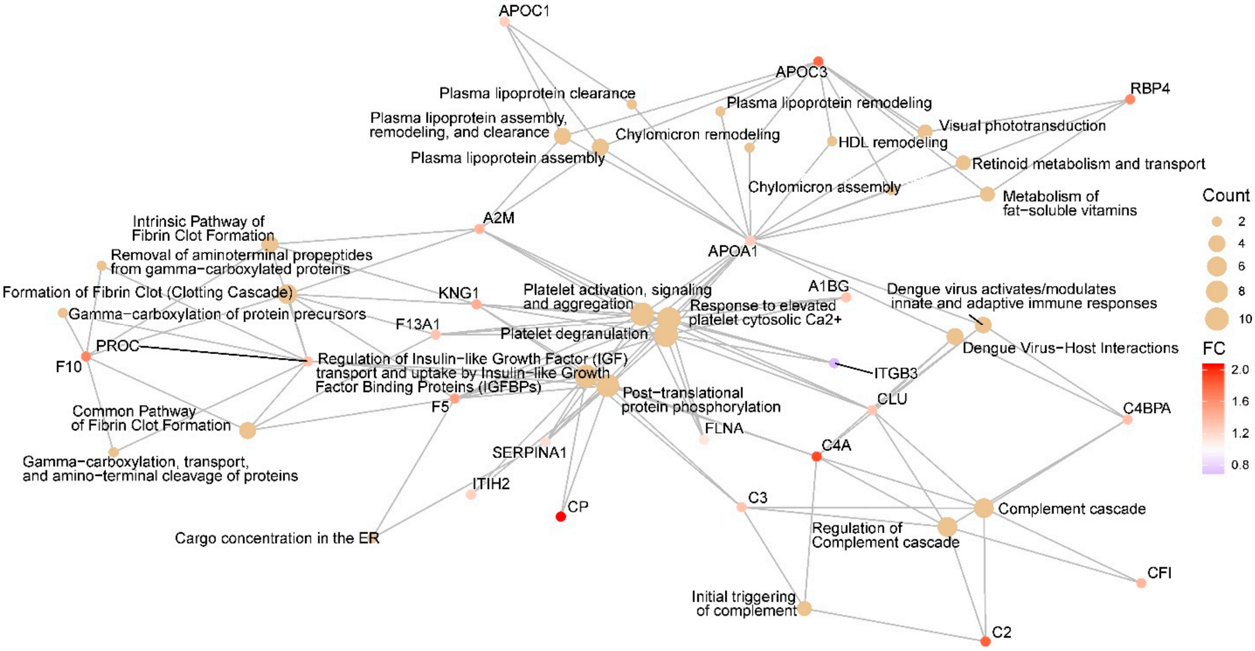
APOC1
APOC3
RBP4
Plasma lipoprotein clearance
Plasma lipoprotein remodeling
Plasma lipoprotein assembly, remodeling, and clearance
Chylomicron remodeling
HDL remodeling
Visual phototransduction
Plasma lipoprotein assembly
Retinoid metabolism and transport
Chylomicron assembly
Metabolism of fat−soluble vitamins
Intrinsic Pathway of Fibrin Clot Formation
A2M
APOA1
Removal of aminoterminal propeptides from gamma−carboxylated proteins
A1BG
Dengue virus activates/modulates innate and adaptive immune responses
Formation of Fibrin Clot (Clotting Cascade)
KNG1
Platelet activation, signaling and aggregation
Response to elevated platelet cytosolic Ca2+
Gamma−carboxylation of protein precursors
F13A1
Platelet degranulation
Dengue Virus−Host Interactions
PROC
Regulation of Insulin−like Growth Factor (IGF) transport and uptake by Insulin−like Growth Factor Binding Proteins (IGFBPs)
ITGB3
F10
F5
Post−translational protein phosphorylation
CLU
Common Pathway of Fibrin Clot Formation
FLNA
C4BPA
C4A
SERPINA1
Gamma−carboxylation, transport, and amino−terminal cleavage of proteins
ITIH2
C3
CP
Complement cascade
Cargo concentration in the ER
Regulation of Complement cascade
CFI
Initial triggering of complement
C2
Count
2
4
6
8
10
FC
2.0
1.6
1.2
0.8

(c)

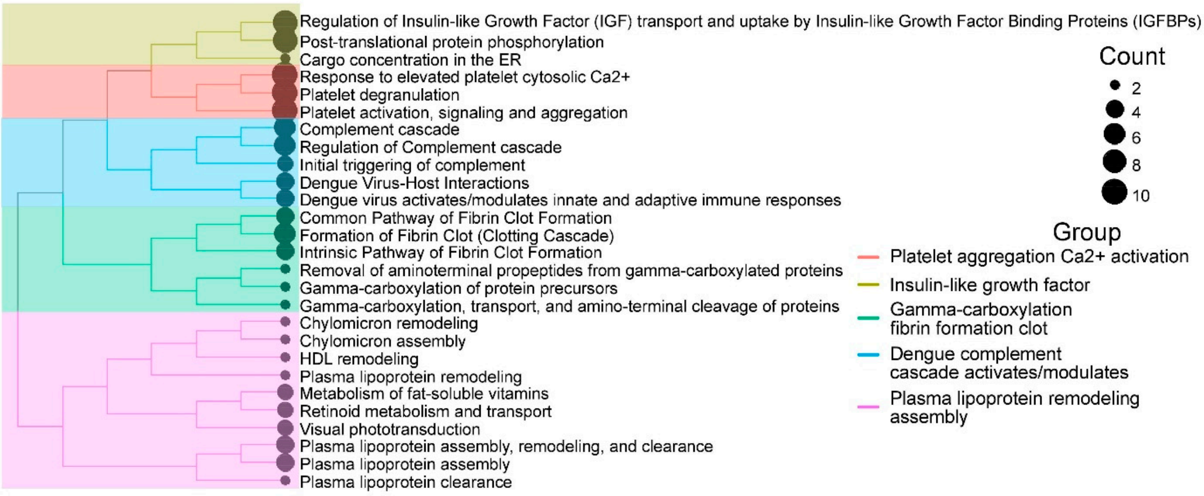
Regulation of Insulin-like Growth Factor (IGF) transport and uptake by Insulin-like Growth Factor Binding Proteins (IGFBPs)
Post-translational protein phosphorylation
Cargo concentration in the ER
Response to elevated platelet cytosolic Ca2+
Platelet degranulation
Platelet activation, signaling and aggregation
Complement cascade
Regulation of Complement cascade
Initial triggering of complement
Dengue Virus-Host Interactions
Dengue virus activates/modulates innate and adaptive immune responses
Common Pathway of Fibrin Clot Formation
Formation of Fibrin Clot (Clotting Cascade)
Intrinsic Pathway of Fibrin Clot Formation
Removal of aminoterminal propeptides from gamma-carboxylated proteins
Gamma-carboxylation of protein precursors
Gamma-carboxylation, transport, and amino-terminal cleavage of proteins
Chylomicron remodeling
Chylomicron assembly
HDL remodeling
Plasma lipoprotein remodeling
Metabolism of fat-soluble vitamins
Retinoid metabolism and transport
Visual phototransduction
Plasma lipoprotein assembly, remodeling, and clearance
Plasma lipoprotein assembly
Plasma lipoprotein clearance
Count
2
4
6
8
10
Group
Platelet aggregation Ca2+ activation
Insulin-like growth factor
Gamma-carboxylation fibrin formation clot
Dengue complement cascade activates/modulates
Plasma lipoprotein remodeling assembly

Figure S1. (a) Pathways statistically significantly enriched with LSIL marker proteins (b) Relationship between statistically significantly enriched pathways and the marker proteins they include. (c) Similarity tree of statistically significantly enriched pathways.

(a)

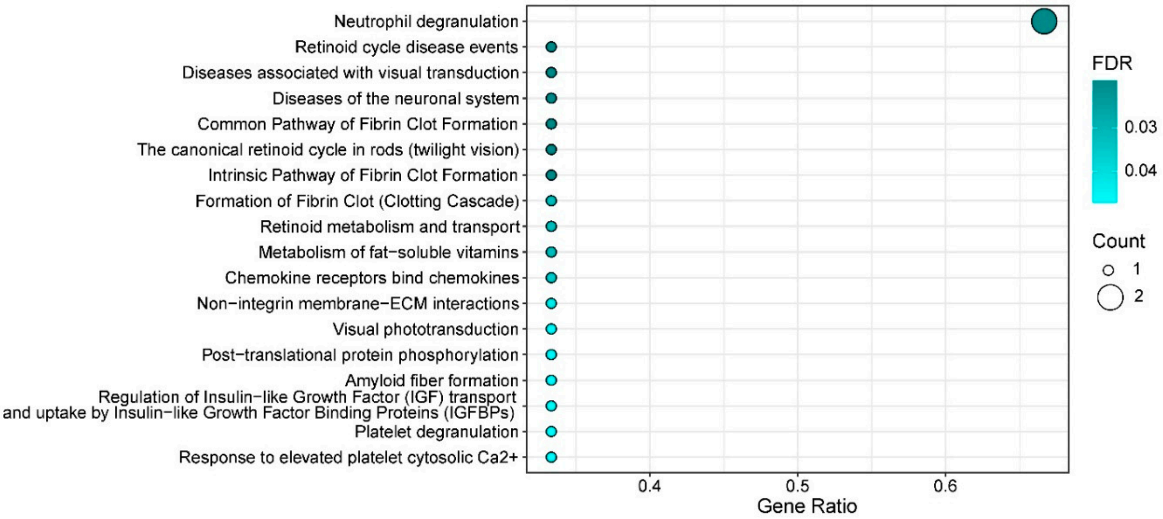

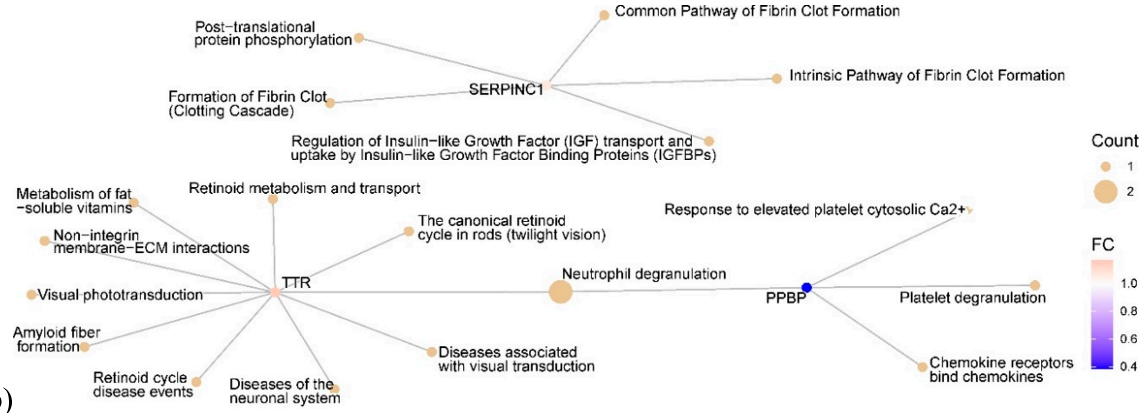

(b)

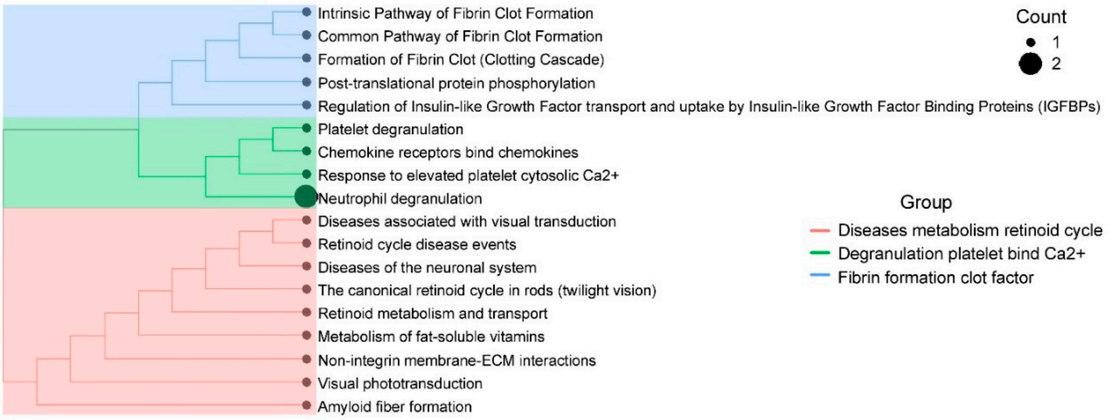

(c)

Figure S2. (a) Pathways statistically significantly enriched with HSIL marker proteins (b) Relationship between statistically significantly enriched pathways and the marker proteins they include. (c) Similarity tree of statistically significantly enriched pathways.

(a)

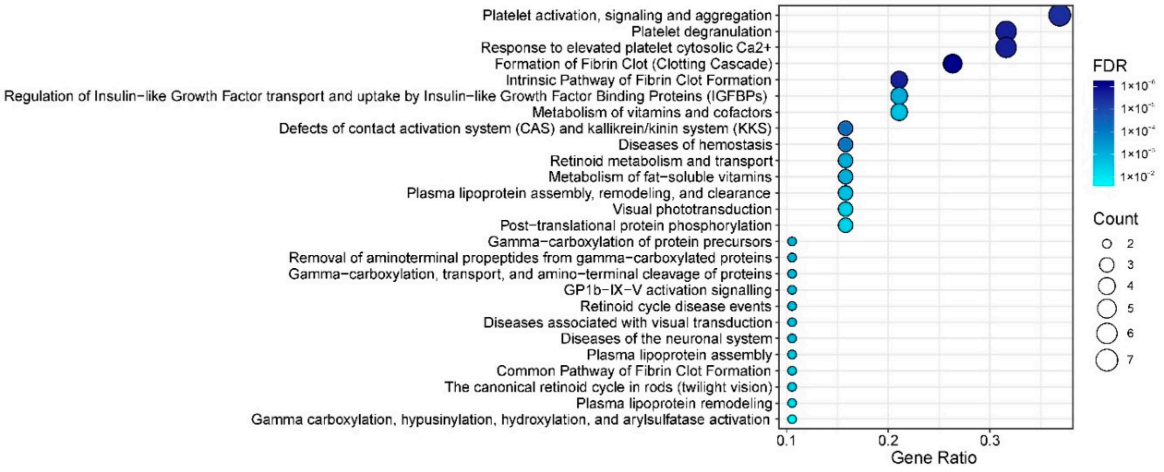

(b)

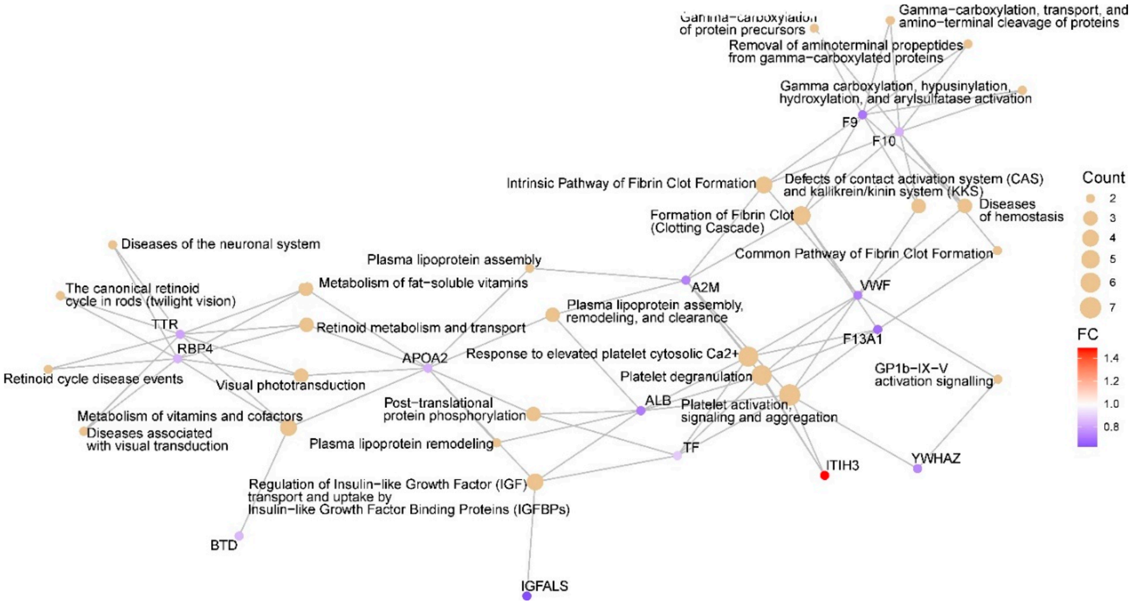

(c)

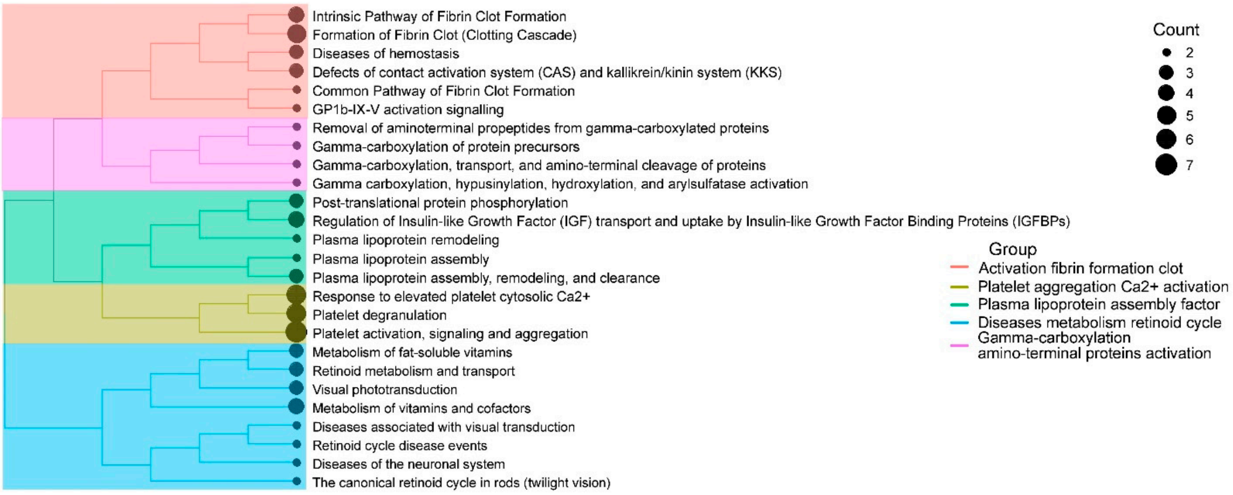

Figure S3. (a) Pathways statistically significantly enriched with cancer marker proteins (b) Relationship between statistically significantly enriched pathways and

the marker proteins they include. (c) Similarity tree of statistically significantly enriched pathways.
